# Association between adiposity and facial aging: results from a Mendelian randomization study

**DOI:** 10.1186/s40001-023-01236-x

**Published:** 2023-09-15

**Authors:** Meiqi Liu, Jingwei Feng

**Affiliations:** Department of Plastic and Cosmetic Surgery, Nanfang Hospital, Southern Medical University, 1838 Guangzhou North Road, Guangzhou, 510515 Guangdong People’s Republic of China

## Abstract

**Background:**

Skin, as a sociologically meaningful interface, has psychological implications different from other organs, particularly in the context of the global population aging. Growing evidence suggests that facial aging is associated with an increased risk of adiposity. Existing research, however, were observational, and while they may find some correlations, it is difficult to simply disentangle non-causal or reverse-causal links because these associations may be confounded or fail to accurately reflect true causative linkages.

**Objectives:**

We conducted a 2-sample Mendelian randomization (MR) study to examine the potential effect of facial aging on the risk of broad obesity and its three major adiposity indicators, including body mass index (BMI), body fat percentage (BF%) and waist circumference (WC).

**Methods:**

Genetic instruments from IEU OpenGWAS project, one of the largest available genome-wide association studies (GWAS) for facial aging (423,999 samples) were used to investigate the relation to broad obesity (32,858 cases, 65,839 controls). Using the inverse-variance weighted (IVW) technique, single nucleotide polymorphisms (SNPs) associated with adiposity indicators (BMI (461,460 samples), BF% (454,633 samples), and WC (462,166 samples)) were investigated in relationship to facial aging. Further sensitivity analyses were performed, including Mendelian randomization-Egger (MR-Egger), weighted median estimates, and leave-one-out analysis, to evaluate the consistency of the results and related potential issues in MR studies.

**Results:**

We identified strong and significant correlations between adiposity and facial aging in the 17 broad obesity-associated SNPs (IVW estimate of odds ratio OR = 1.020, 95% CI 1.010–1.029, *P* = 7.303e − 05), 458 BMI-associated SNPs (IVW estimate of odds ratio OR = 1.047, 95% CI 1.0357–1.058, *P* = 1.154e − 16),for the 395 BF%-associated SNPs (OR = 1.056, 95%CI 1.040–1.072,P = 7.617e − 12), or for the 374 WC-associated SNPs (OR = 1.072, 95% CI 1057–1.087,P = 1.229e − 23). A range of complementary methodologies have been employed to evaluate horizontal pleiotropy and related potential caveats occurring in MR research.

**Conclusions:**

Using Mendelian randomization as an alternative approach to investigate causality, we found a causal relationship between adiposity and facial aging, which was statistically strong and significant.

**Supplementary Information:**

The online version contains supplementary material available at 10.1186/s40001-023-01236-x.

## Key Messages

- Using a two-sample Mendelian randomization, we found a causal relationship between adiposity (and its three major adiposity indicators, including body mass index, body fat percentage and waist circumference) and facial aging, which was statistically strong and significant.
- Among the three adiposity indicators, WC had a better casual association with facial aging, which indicated that subcutaneous fat may present a major contribution in facial aging compared with visceral fat.
- More detailed causal relationship was revealed in negative results of subsequent multivariable MR analysis (three major adiposity indicators with facial aging) and two sample MR analysis between visceral/subcutaneous adipose tissue and facial aging.

**Supplementary Information:**

The online version contains supplementary material available at 10.1186/s40001-023-01236-x.

## Introduction

Adiposity is a worldwide major public health problem with an alarmingly increasing prevalence over the past 2 decades. Forty-two percent of adults in the United States are obese, and a total of 1.9 billion adults worldwide are overweight or obese, which continue to climb, represent a major health and economic burden [1]. During obesity, skin-resident and systemic adipose tissue derives secreted factors and increases inflammation and has detrimental impact on facial aging [2–4]. The consequences of obesity in the skin are underestimated. Increased body mass index puts great disserve to skin physiology, skin barrier, collagen structure, and wound healing process, triggering the possibility of skin cancer [5]. Skin, as a sociologically meaningful interface, has psychological implications different from other organs, which makes facial rejuvenation and skin health care particularly attractive in the context of the global population aging. With the popularization of health care knowledge and the improvement of living conditions, the life span of the general population has extended and the global population has aged, which leads to a major paradigm shift in the focus of medical research to preventing and managing aging and optimizing the overall healthy life span [6, 7]. The society is paying increasing attention to the treatment and early intervention of skin aging.

Growing data suggest that facial aging is linked to obesity, lowering quality of life, and compromising mental health [8, 9]. In 2007, Yosipovitch et al. highlighted the association between adiposity and dermatologic conditions [10]. Subsequently, systematic reviews and meta-analyses have validated these putative correlations between adiposity (mostly BMI used as an indicator) and face aging [6, 11]. However, previous studies are based on observational epidemiological designs, which are prone to reverse causation and unmeasured confounding [12]. Specifically, the independent roles of adipose tissue from different depots in the relationship between adiposity and facial aging are still unclear, which is crucial to improve the rejuvenation modalities. Mendelian randomization (MR) provides an alternative approach to investigate causality by using genetic variants as instrumental variables and thereby accounting for observational study bias [13–15]. Here, we provide an MR analysis of the relationship between face aging and obesity along with a more precise indicator.

## Methods

UK Biobank is a large-scale biomedical database and research resource that contains detailed genetic and health data from over half a million UK individuals [16]. In UK Biobank, Participants were recruited from National Health Service central registries across the UK, with provided informed permission and the Northwest Multicenter Research Ethics Committee granted ethical approval. Considering the genome-wide association studies (GWAS) used to identify genetic instruments were predominantly based on European samples, our analysis exclusively included European subjects.

*Adiposity* measures were ascertained following standardized protocols [6, 17]. Weight was measured without shoes and heavy outer clothing and BF% were measured via a Tanita BC-418 MA body composition analyser. Height was measured with a Seca 202 height measure, and waist circumferences were obtained with a tape measure [6, 17]. BMI was computed using National Institutes of Health standard (https://www.nhlbi.nih.gov/health/educational/lose_wt/BMI/bmicalc.htm). BF% and WC’s GWAS data were output from GWAS pipeline using Phesant derived variables from UK Biobank [18]. As genetic instruments for BMI, BF%, and WC, we employed single nucleotide polymorphisms (SNPs) detected from the largest European descendant GWAS to date (458, 395, 374 near-independent SNPs, respectively) [17, 19].

*Facial aging* was conducted using data from the population-based UK Biobank. This deposit from which facial aging domain was obtained, provides full details of the genome-wide association study (GWAS) pipeline developed by the MRC-IEU for the full UK Biobank (version 3, March 2018) genetic data [20]. The method of this domain to determine perceived age based on questionnaire has been described previously [21, 22]. Subjective evaluations of facial aging were measured by the question: do people say that you look younger than you are, older than you are, about your age, do not know, and prefer not to answer?

### MR analysis

The data on the necessary variables and the facial aging GWAS were harmonized by the separate chromosomes and positions. The five MR methods applied in the research have been described previously [23, 24]. The random-effects inverse-variance weighted (IVW) method was used as the primary estimator for the MR analysis [25], for it has a higher statistical power with the assumption that all SNPs are valid instrumental variables. The weighted median approach [26], MR-Egger regression [27], weighted mode method [28] and simple mode method were used as reciprocal analysis. To investigate whether any single SNP had a disproportionate effect on the overall results, IVW analyses were re-performed leaving out SNPs once a time. To investigate if the SNPs affected facial ageing but not through adiposity, horizontal pleiotropy analysis was applied and identified SNPs of heterogeneity were extracted separately for literature background check. The causal estimates would be regarded as heterogeneous if the *p* value for Cochran’s *Q* test was above 0.05 and *I*^2^ was above 0.25. Scatter plots, leave-one-out analysis plots, forest plots and funnel plots were created to visually assess the results.

All statistical analyses were conducted in R 3.6 and TwoSampleMR package [29]. R version 3.4.0 and RStudio 2022.07.2 both for MacOS, were used for all statistical analyses. The weighted median method was used to supplement the MR-Egger, in order to gain a further dependable estimation of the unproductive effect.

The present study only used publicly available summary-level statistics. No individual-level data were analyzed. Ethical approval therefore was not required.

### Sensitivity analyses

Sensitivity analyses were applied, including heterogeneity and pleiotropy analysis, to rule out the potential that these SNPs influenced the Mendelian randomization studies. A leave-one-out analysis was also completed to determine whether any SNP-driven relationships existed.

## Result

To establish the association between the SNP’s effect on the exposure (extensive adiposity and three adiposity markers) and the SNP’s effect on the outcome (facial aging), we contrasted the traditional IVW analysis with the MR egger analysis for potential horizontal pleiotropic correction.

### Primary analysis for broad obesity

**Fig.1:**
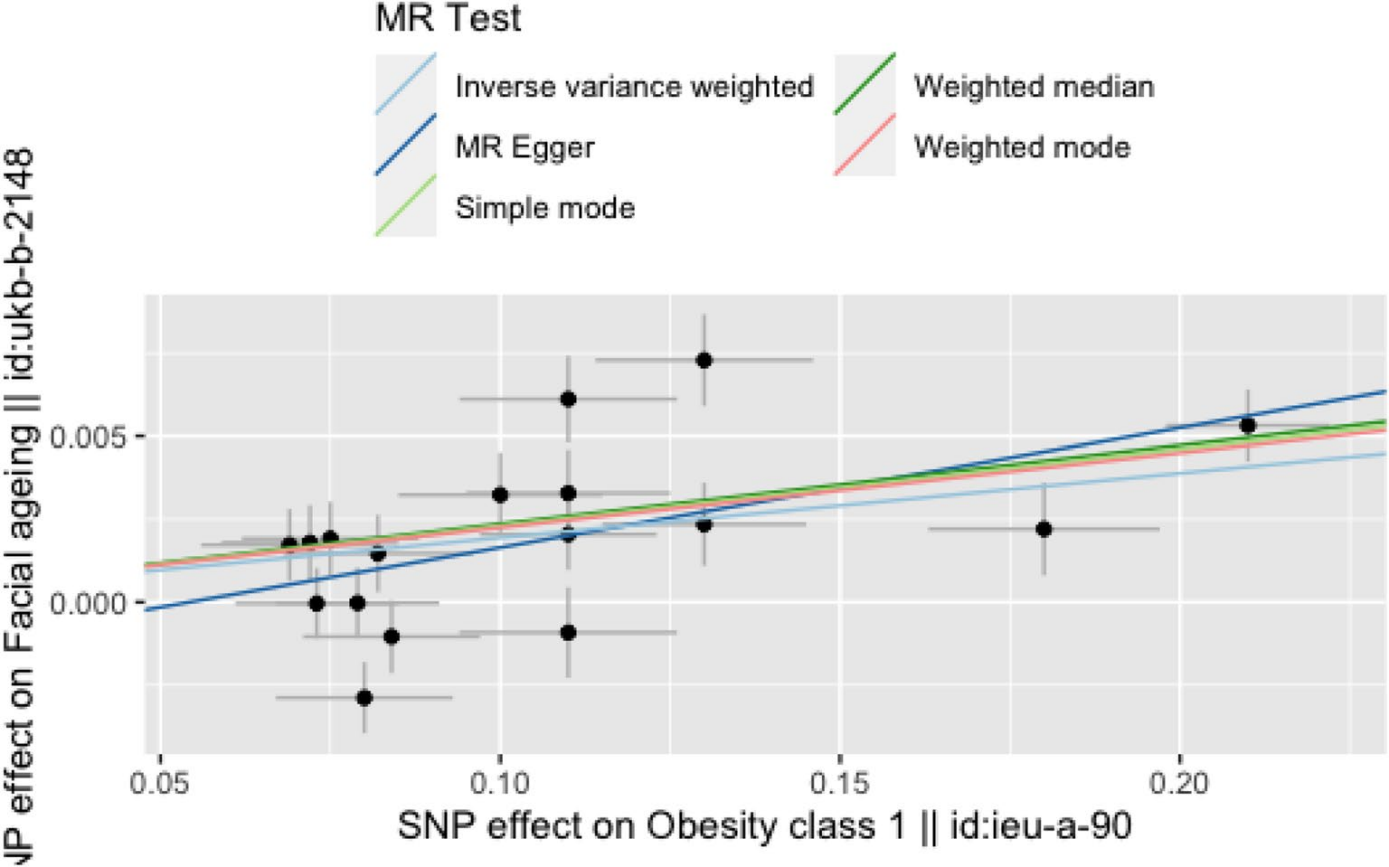
Scatter plot for the effects of SNPs on broad obesity and facial aging. IVW, inverse-variance weighted; MR, Mendelian randomization. The horizontal axis represents the effects of each genetic variant on broad obesity, and the vertical axis denotes the effects of each genetic variant on facial aging. The grey lines around the solid black points are the corresponding confidence intervals for the effects. The slopes of solid lines represent the estimates from *IVW* weighted median, weighted mode, single mode and MR-Egger regression analyses

**Fig.2:**
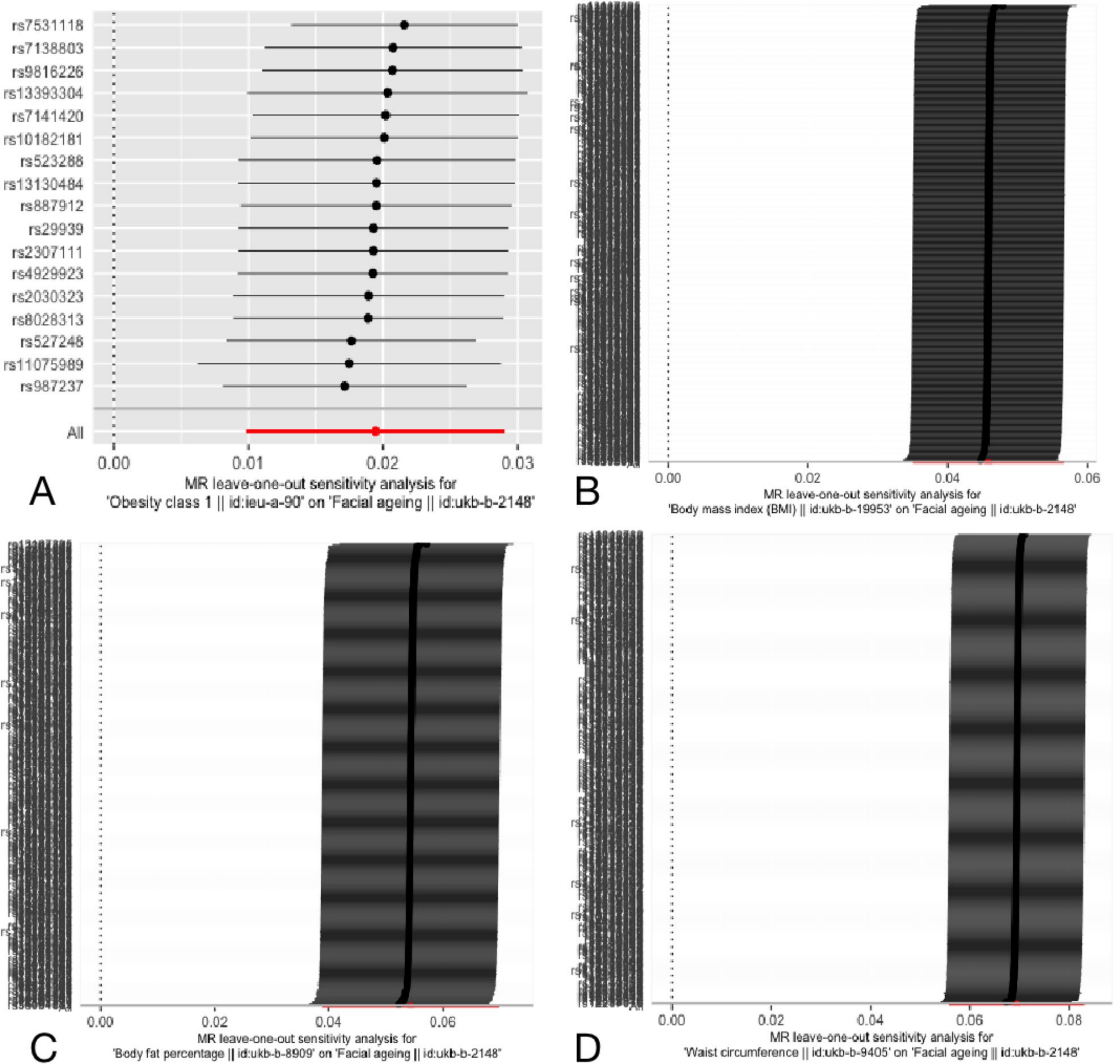
Leave-one-out analysis for the estimates for broad obesity (**A)**, BMI (**B)**, BF% (**C)** and WC (**D**) on facial aging. More details of the vertical coordinate displayed Additional file 3

A significant association was found with facial aging for the 17 broad obesity-associated SNPs (IVW estimate of odds ratio OR = 1.020, 95% CI 1.010–1.029, *P* = 7.303e − 05). There was some evidence of heterogeneity in the SNP effects (Table 2). MR-PRESSO analysis identified five influential outlier which were the TFAP2B variant, rs987237 (outlier test *p*-value < 0.017), the faim2 variant rs7138803 (outlier test p-value = 0.153), the ETV5 variant rs9816226 (outlier test *p*-value = 0.493), a NEGR1 obesity locus rs7531118 (outlier test *p*-value < 0.017), and rs527248 (outlier test *p*-value = 0.017) which were also shown to be a clear outlier in both scatter and leave-one-out plots (Figs. 1 and 2). These genes all performed a strong linkage with adiposity. Therefore, we retained these SNPs without eliminating these outliers, considering this may be caused by the ethnic group and environment (such as sunshine time, air humidity and air quality) differences between representative samples in the databases of UK Biobank and IEU OpenGWAS.

### Secondary analysis for adiposity indicators

**Fig.3:**
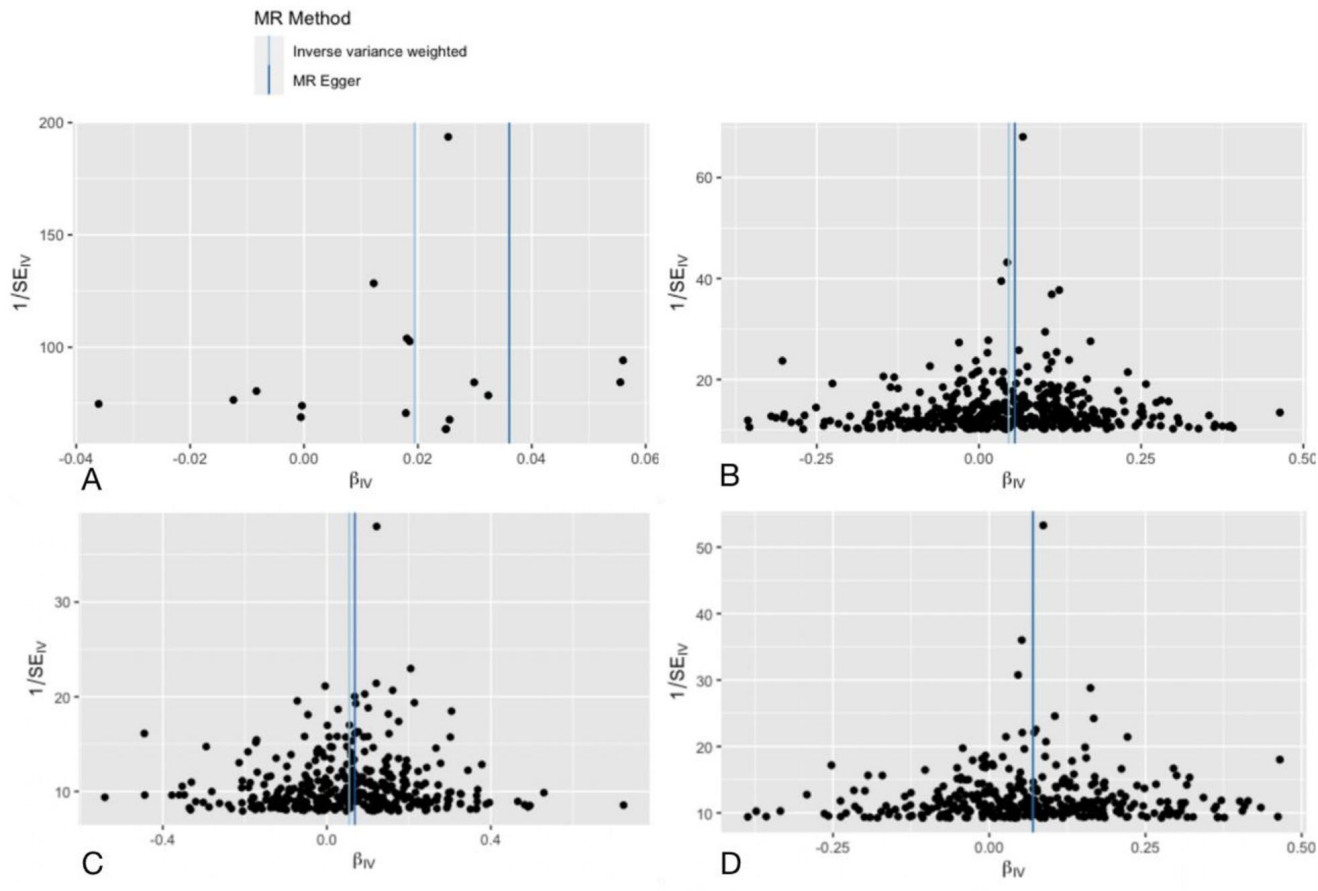
Funnel plot for the SNPs for broad obesity **A**, BMI (B), BF% **C** and WC **D** on facial aging

**Fig.4:**
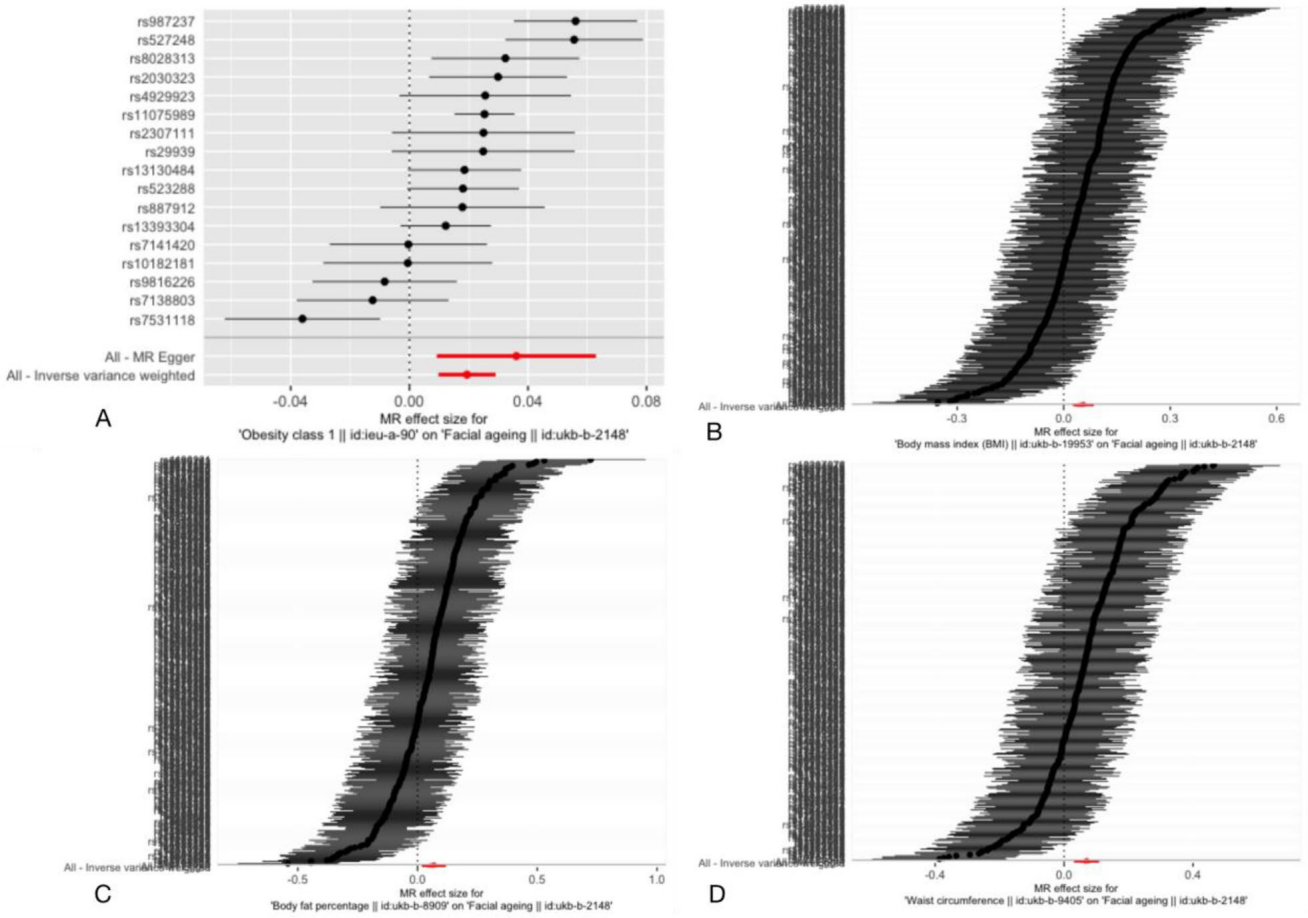
Forest plot for the estimates for broad obesity (**A)**, BMI (**B)**, BF% (**C)** and WC (**D)** on facial aging. More details of the vertical coordinate displayed Additional file 3

**Fig.5:**
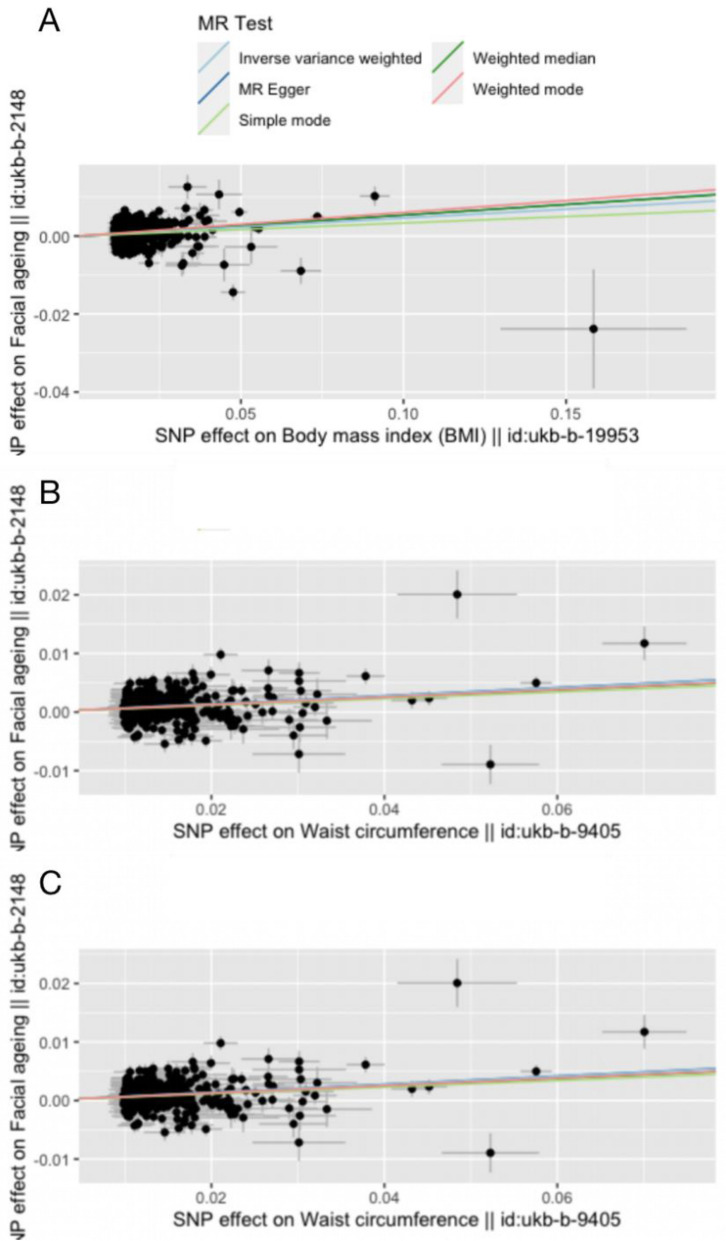
Scatter plot for the effects of SNPs on BMI (**A)**, BF% (**B)** and WC (**C)** and facial aging

Tables 1, 2, 3 and Figs. 2, 3 reveal the results. Significant and strong associations with facial aging were also reported for the 458 BMI-associated SNPs, for the 395 BF%-associated SNPs, and for the 374 WC-associated SNPs (Table 1, Figs. 4, 5). At a type one error of 5%, we had ≥ 95% power to detect a causal association of an odds ratio OR > 1 with BMI, BF% and WC. We detected OR > 1.05 for BF% and OR > 1.06 for WC with a power of > 95% (Table 1). Applying standard IVW analysis, we found evidence for effects of genetically instrumented facial ageing on BMI (OR = 1.047, 95% CI 1.036–1.058, P = 1.154e − 16)), BF% (OR = 1.056, 95%CI 1.040–1.072, *P* = 7.617e − 12), and WC (OR = 1.072, 95% CI 1057–1.087, *P* = 1.229e − 23), respectively (Table 1, Fig. 5). All five MR methods (IVW, weighted median, weighted mode, single mode, and MR-Egger) that were investigated showed nearly identical direction of the effect (Table 1).

**Table 1:** Mendelian randomization (MR) estimates for the relationship between genetically instrumented broad obesity, BMI, BF%, WC and facial aging

Exposure	Method	Outcome: facial aging (P < 5 × 10–4)		
		OR	95%CI	*p*-value
Broad obesity	Inverse variance weighted	1.020	1.010, 1.029	7.303e-05
	MR-Egger regression	1.037	1.009, 1.065	1.872e-02
	Weighted median	1.024	1.016, 1.032	4.661e-09
	Simple mode	1.023	1.010, 1.037	3.131e-03
	Weighted mode	1.023	1.014, 1.032	1.722e-04
BMI	Inverse variance weighted	1.047	1.036, 1.058	1.154e-16
	MR-Egger regression	1.057	1.026, 1.088	2.626e-04
	Weighted median	1.055	1.043, 1.068	6.407e-20
	Simple mode	1.034	0.990, 1.080	1.407e-01
	Weighted mode	1.063	1.037, 1.089	1.437e-06
BF %	Inverse variance weighted	1.056	1.040, 1.072	7.617e-12
	MR-Egger regression	1.071	1.019, 1.126	7.679e-03
	Weighted median	1.066	1.048, 1.083	9.156e-15
	Simple mode	1.065	1.006, 1.127	3.017e-02
	Weighted mode	1.078	1.030, 1.128	1.385e-03
WC	Inverse variance weighted	1.072	1.057, 1.087	1.229e-23
	MR-Egger regression	1.072	1.031, 1.115	4.842e-04
	Weighted median	1.065	1.049, 1.081	5.379e-16
	Simple mode	1.059	1.007, 1.113	2.529e-02
	Weighted mode	1.065	1.036, 1.096	1.059e-05

**Table 2:** Heterogeneity analysis for the relationship between genetically instrumented broad obesity, BMI, BF%, WC and facial aging

Exposure	Methods	Outcome: facial aging (*P* < 5 × 10–4)	
		*Q*	*Q*-*p* value
Broad obesity	Inverse variance weighted	57.770	1.237e-06
	MR-Egger regression	51.940	5.780e-06
BMI	Inverse variance weighted	1305.159	1.425e-86
	MR-Egger regression	1303.805	1.295e-86
BF %	Inverse variance weighted	1140.238	1.198e-76
	MR-Egger regression	1140.236	9.660e-77
WC	Inverse variance weighted	1031.215	8.828208e-66
	MR-Egger regression	1031.214	5.208e-66

**Table 3:** Pleiotropy analysis for the relationship between genetically instrumented broad obesity, BMI, BF%, WC and facial aging

Exposure	Methods	Outcome: facial aging (*P* < 5 × 10–4)	
		se	*P* value
Broad obesity	Pleiotropy test	0.0015	0.214
BMI		0.0002718907	0.500
BF%		0.0003466899	0.5629983
WC		0.0003062123	0.9803325

All of the evaluated genetic factors for adiposity were found to be separately linked with face aging after multiple testing adjustment. Genetic pleiotropy does in fact cause causal bias, as shown by the clear statistical significance shown using MR-Egger regression analysis (Table 3). Low heterogeneity in the IVW estimate and MR-Egger regression was observed for all outcomes (BMI: *Q*-pval ≤ 1.425e − 86; BF% Q-pval ≤ 1.198e − 76; WC: Q-pval ≤ 8.828e − 66, Table 2). Our pleiotropy methods also indicated no relations of facial ageing to BMI, BF% or WC (0.500 ≤ p ≤ 0.980, Table 3). For further sensitivity analyses, we carried out leave-one-out analysis to assess whether the IVW estimate was driven by an individual SNP and performed positive and negative control outcome analyses. The leave-one-out analysis revealed that a single genetic marker was primarily responsible for the linked signals (Fig. 2).

## Discussion

In the present study, for the first time, we examined the association of genetically predicted adiposity and facial aging in a large population-based cohort, the UK Biobank (as well as IEU OpenGWAS project applied in supplementary materials). By leveraging several MR estimation approaches, we found that longer genetically predicted obesity was associated with a more severe likelihood of facial aging. Our study, corroborating previous observational and experimental studies, provides further evidence to support a causal role of adiposity in facial aging. And to the best of our knowledge, no studies have used population-based cohorts to investigate the topic.

The difficulty in measuring face aging is one reason why there are so few research on this topic at the population level. For instance, although three-dimensional human face morphology evaluation devices [35], among others [36], can deliver objective and comprehensive characteristics for facial aging, aging researchers have not had widespread access to them due to their impracticability and high cost. A subjective evaluation of facial aging may prove helpful in overcoming the difficulties associated with measuring face aging and in maximizing the use of questionnaire-based tools [37]. That’s why we finally chose to use questionnaire-based measurements from the UK Biobank [38, 39]. The UK Biobank and IEU OpenGWAS projects’ high sample sizes have the potential to allay worries about measurement error for the evaluation of face aging based on questionnaires, which is a benefit of this sort of data.

Simultaneously, when discussing the potential risks of adiposity, people frequently refer to the involvement of adipose tissue. SAT plays an essential role in the regulation of skin homeostasis and rejuvenation [40, 41]. Laboratory studies showed that age-dependent regulatory cells (ARCs) in SAT increase in abundant after middle age and display high levels of pro-inflammatory markers, which may accelerate facial aging [42]. However, as limited publicly available databases, the facial ageing’s casualty with subcutaneous adipose tissue (SAT) cannot be detected with only two SNPs harvested from GWAS (Additional file 2).

Then we shifted our gaze to visceral adipose tissue (VAT). An UK Biobank-based MR research identified a link between genetically determined VAT accumulation and longevity, showing that visceral adiposity may be detrimental to systemic aging [43]. Based on this, we investigated VAT’s effects on the more localized facial aging. More detailed causal relationship was revealed in the negative results of multivariable MR analysis and two sample MR analysis between VAT and facial aging (Additional file 1). Among the three adiposity indicators, WC has a better casual association with facial aging (Additional file 1: Table S1) Then, a two sample MR analysis was implemented between VAT and facial aging. We observed that the OR per SD increase of VAT on facial ageing using the IVW method was 1.047 (95% CI − 0.002, 0.093) and *P*-value is greater than 0.05 among the five MR methods tested (Additional file 1: Table S2).

The primary and secondary effects of SAT and VAT on facial aging have not yet been verified. Although VAT has no anatomic advantage over SAT, considering that the increase of WC is more closely related to the accumulation of VAT, we suggested that the excessive VAT accumulation might also be involved in the aggravation of facial aging through multiple pathophysiological processes [46]. One the other hand, several researchers considered that white adipose tissue (WAT) in SAT can be a beneficial therapeutic target for facial aging. Subcutaneous WAT have a thick ‘skin’ confronting with the down-regulating behavior of aging-regulated RNA [47, 48]. Among a pairwise age group comparisons, only a few immune- and inflammation-associated genes are found to be differentially regulated in the skin compared to the brain and blood, indicating that subcutaneous WAT performs a better immunomodulatory capacity with typical inflammaging signs [49].

In addition, factors affecting facial aging are also very diverse. Novel results revealed a detrimental effect of heavier smoking on facial aging [50]. Another nonlinear analysis indicated a potential threshold relationship between alcohol intake and telomere length [51]. But there is still no MR evidence showing alcohol consumption or other unhealthy lifestyles have any direct causality with facial aging. There is also little statistical evidence suggesting whether factors of low-quality life (like poverty, disability, long-term UV exposure or air pollution exposure), and mental health (including depression and anxiety) trigger facial aging. We will continue to conduct studies on these topics.

## Limitations

Despite the advantages of the large sample size, our study is prone to several limitations. First and foremost, in this study we performed reliable and meaningful estimations using a variety of MR methods. Adiposity can be used as a mediator to further investigate the underlying molecular causes of face aging. Simultaneously, it is also necessary to analyze data in more nuanced manners, such as grouping data according to race, gender and living habits, or applying more complex MR models to verify their direct or indirect associations. Second, although we have assumed a linear relationship between adiposity and facial aging in this study, we still do not know whether this is a direct linear causal relationship or indirect nonlinear relationship. Further research needs to be conducted by applying more complex MR models to verify their direct or indirect correlations. Third, considering there is few basic research with high hierarchy of evidence revealing the relationship between adiposity and facial aging or between subcutaneous adipose tissue and facial aging, our present findings need further replication in multicenter cohorts and basic research contained high-throughput sequencing analysis of human samples. Last but not least, as previously stated, the UK Biobank and IEU OpenGWAS research quantified face age using a single query, which could lead to measurement error. Future research is encouraged in order to create a more comprehensive questionnaire-based approach for measuring diverse aspects of facial aging. In this study, we performed reliable and meaningful estimations using a variety of MR methods. Adiposity can be used as a mediator to further investigate the underlying molecular causes of face aging. Simultaneously, it is also necessary to analyze data in more nuanced manners, such as grouping data according to race, gender and living habits, or applying more complex MR models to verify their direct or indirect associations.

## Conclusions

Our study provides evidence that obesity-related risk drives facial ageing. Using Mendelian randomization, a greater adiposity genetic susceptibility was causally related to facial ageing when applying the genetic instruments from UK Biobank and IEU OpenGWAS project. Given the rapidly rising burden of obesity across the world, our findings have important implications for global health and esthetic skin rejuvenation.

### Supplementary Information

**Additional file 1: Table S1.** Multivariate Mendelian randomization (MR) estimates for the relationship between genetically instrumented board obesity, BMI, BF%, WC and facial aging. **Table S2.** Mendelian randomization (MR) estimates for the relationship between genetically instrumented VAT and facial aging. **Table S3.** Heterogeneity analysis for the relationship between genetically instrumented VAT and facial aging. **Table S4.** Pleiotropy analysis for the relationship between genetically instrumented VAT and facial aging. **Fig. S1.** Scatter plot for the effects of SNPs on VAT and facial aging. **Fig. S2.** Leave-one-out analysis for the estimates for VAT on facial aging. **Fig. S3.** Forest plot for the estimates for VAT on facial aging. **Fig. S5.** Funnel plot for the SNPs for VAT on facial aging

**Additional file 2: Fig. S1.** Scatter plot for the effects of SNPs on SAT and facial aging. **Fig. S2.** Forest plot for the estimates for SAT on facial aging.

**Additional file 3:** The original data of Leave-one-out analysis and Forest plots.

## Data Availability

The data that support the findings of this study are available from UK Biobank project site and IEU OpenGWAS project site, subject to successful registration and application process. Specifically the data of facial ageing, BMI, BF% and WC are from UK Biobank and the data of broad obesity is from the IEU OpenGWAS. Further details can be found at https://www.ukbiobank.ac.uk/ or https://gwas.mrcieu.ac.uk/.

## Abbreviations

MR: Mendelian randomization
BMI: Body mass index
BF%: Body fat percentage
WC: Waist circumference
GWAS: Genome-wide association studies
SNPs: Single nucleotide polymorphisms
IVW: Inverse-variance weighted
MR-Egger: Mendelian randomization-Egger
SD: Standard deviation
SE: Standard error
OR: Odds ratio
CI: Confidence interval
VAT: Visceral adipose tissue
SAT: Subcutaneous adipose tissue
ARCs: Age-dependent regulatory cells
WAT: White adipose tissue
