# Supplementary material for: Association between adiposity and facial aging: results from a Mendelian randomization study
Source: Eur J Med Res. 2023 Sep 15;28:350. doi: 10.1186/s40001-023-01236-x (PMC10503104; doi:10.1186/s40001-023-01236-x)
Supplement: Supplementary file 1 — Additional file 1: Table S1. Multivariate Mendelian randomization (MR) estimates for the relationship between genetically instrumented board obesity, BMI, BF%, WC and facial aging. Table S2. Mendelian randomization (MR) estimates for the relationship between genetically instrumented VAT and facial aging. Table S3. Heterogeneity analysis for the relationship between genetically instrumented VAT and facial aging. Table S4. Pleiotropy analysis for the relationship between genetically instrumented VAT and facial aging. Fig. S1. Scatter plot for the effects of SNPs on VAT and facial aging. Fig. S2. Leave-one-out analysis for the estimates for VAT on facial aging. Fig. S3. Forest plot for the estimates for VAT on facial aging. Fig. S5. Funnel plot for the SNPs for VAT on facial aging [file 40001_2023_1236_MOESM1_ESM.docx]

Additional file 1

Table 1. Multivariate mendelian randomization (MR) estimates for the relationship between genetically instrumented board obesity, BMI, BF%, WC and facial aging.

| Exposure | Method | Outcome: Facial aging (P < 5 × 10–4) | |
| --- | --- | --- | --- |
|  |  | 95%CI | p-value |
| BMI | Egger | -0.066, 0.037 | 0.577 |
|  | Inverse variance weighted | -0.068, 0.026 | 0.382 |
|  | LASSO | -0.027, 0.039 | 0.713 |
|  | Multivariable median method | -0.028, 0.059 | 0.484 |
|  | MVMR | - | 0.125 |
| BF % | Egger | -0.072, 0.030 | 0.423 |
|  | Inverse variance weighted | -0.074, 0.022 | 0.291 |
|  | LASSO | -0.016, 0.049 | 0.320 |
|  | Multivariable median method | -0.030, 0.074 | 0.400 |
|  | MVMR | - | 0.718 |
| WC | Egger | 0.044, 0.173 | 0.001 |
|  | Inverse variance weighted | 0.046, 0.175 | 0.001 |
|  | LASSO | 0.002, 0.089 | 0.038 |
|  | Multivariable median method | -0.032, 0.093 | 0.337 |
|  | MVMR | - | 0.113 |

Table 2. Mendelian randomization (MR) estimates for the relationship between genetically instrumented VAT and facial aging.

| Exposure | Method | Outcome: Facial aging (P < 5 × 10–4) | | |
| --- | --- | --- | --- | --- |
|  |  | OR | 95%CI | p-value |
| VAT | Inverse variance weighted | 1.047 | -0.002, 0.093 | 0.060 |
|  | MR-Egger regression | 0.936 | -0.500, 0.367 | 0.784 |
|  | Weighted median | 1.032 | -0.001， 0.065 | 0.060 |
|  | Simple mode | 1.036 | -0.019， 0.088 | 0.271 |
|  | Weighted mode | 1.033 | -0.013， 0.077 | 0.232 |

Table 3. Heterogeneity analysis for the relationship between genetically instrumented VAT and facial aging.

| Exposure | Method | Outcome: Facial aging (P < 5 × 10–4) | |
| --- | --- | --- | --- |
|  |  | Q | Q-pval |
| VAT | Inverse variance weighted | 17.178 | 0.00178 |
|  | MR-Egger regression | 15.806 | 0.00124 |

Table 4. Pleiotropy analysis for the relationship between genetically instrumented VAT and facial aging.

| Exposure | Method | Outcome: Facial aging (P<5×10–4) | |
| --- | --- | --- | --- |
|  |  | se | pval |
| VAT | Pleiotropy  Test | 0.0095 | 0.645 |


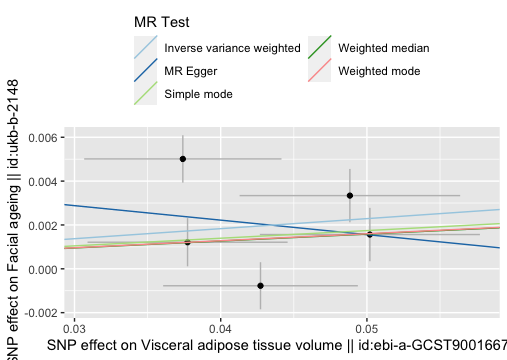


Fig.1 Scatter plot for the effects of SNPs on VAT and facial aging.


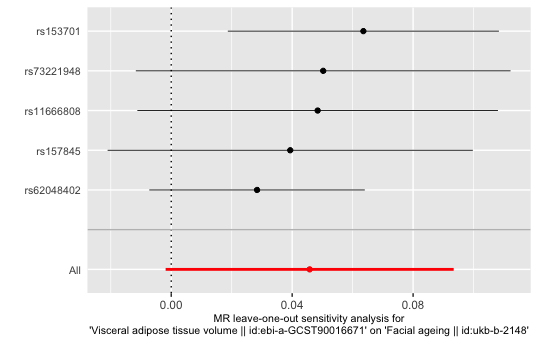


Fig.2 Leave-one-out analysis for the estimates for VAT on facial aging.


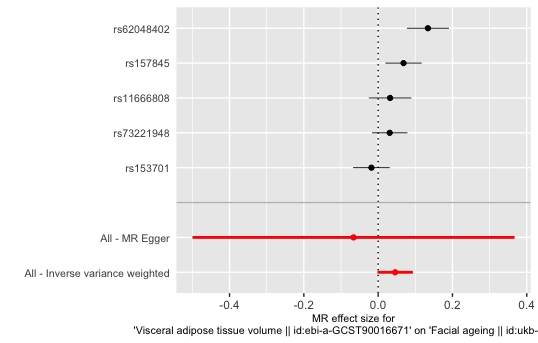


Fig.3 Forest plot for the estimates for VAT on facial aging.


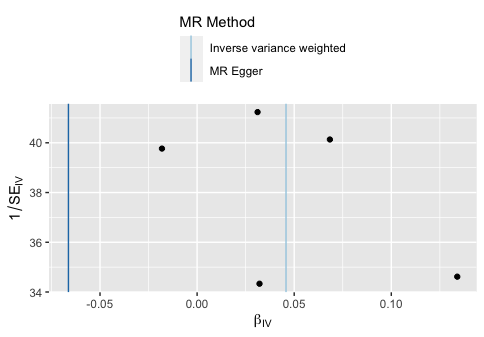


Fig.4 Funnel plot for the SNPs for VAT on facial aging.
