# Supplementary material for: Association between adiposity and facial aging: results from a Mendelian randomization study
Source: Eur J Med Res. 2023 Sep 15;28:350. doi: 10.1186/s40001-023-01236-x (PMC10503104; doi:10.1186/s40001-023-01236-x)
Supplement: Supplementary file 2 — Additional file 2: Fig. S1. Scatter plot for the effects of SNPs on SAT and facial aging. Fig. S2. Forest plot for the estimates for SAT on facial aging. [file 40001_2023_1236_MOESM2_ESM.docx]

Additional file 2


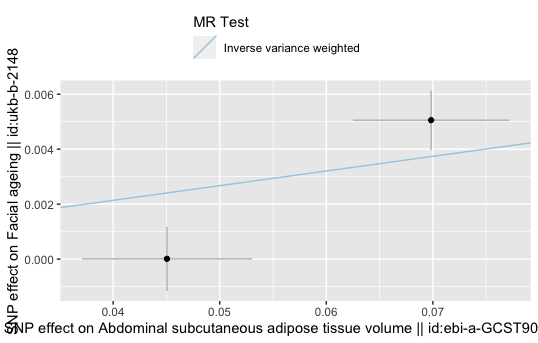


Fig.1 Scatter plot for the effects of SNPs on SAT and facial aging.


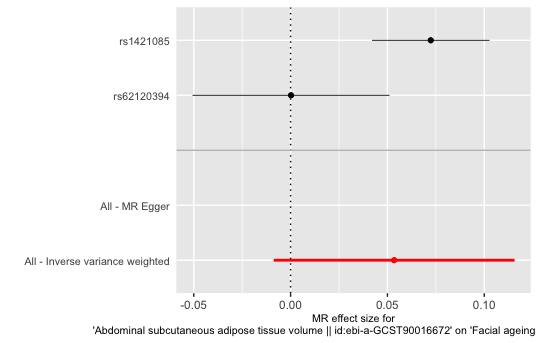


Fig.2 Forest plot for the estimates for SAT on facial aging.
